# Microbiological trends and mortality risk factors of central line-associated bloodstream infections in an academic medical center 2015–2020

**DOI:** 10.1186/s13756-023-01338-5

**Published:** 2023-11-19

**Authors:** Marwan Jabr Alwazzeh, Amani Alnimr, Samia A. Al Nassri, Sara M. Alwarthan, Mashael Alhajri, Bashayer M. AlShehail, Mahdi Almubarak, Nada S. Alghamdi, Haytham A. Wali

**Affiliations:** 1 Infectious Disease Division, Department of Internal Medicine, College of Medicine, Imam Abdulrahman Bin Faisal University, King Fahad Hospital of the University, Dammam, Al-Khobar, Saudi Arabia; 2 Department of Microbiology, King Fahad Hospital of the University, Al-Khobar, Saudi Arabia; 3 Infection Control Unit, King Fahad Hospital of the University, Al-Khobar, Saudi Arabia; 4 Pharmacy Practice Department, College of Clinical Pharmacy, Imam Abdulrahman Bin Faisal University, Dammam, Saudi Arabia; 5 Department of Internal Medicine, College of Medicine, Imam Abdulrahman Bin Faisal University, Dammam, Saudi Arabia; 6 Department of Microbiology, College of Medicine, Imam Abdulrahman Bin Faisal University, Dammam, Saudi Arabia; 7 Department of Pharmacy Practice, College of Clinical Pharmacy, King Faisal University, Al- Ahsa, Saudi Arabia

**Keywords:** Central line Infections, Risk factors, Antibiotic resistance, Mortality

## Abstract

**Background:**

Despite tremendous efforts to prevent central line-associated bloodstream infections, they still remain life-threatening complications among hospitalized patients with significant morbidity and mortality worldwide. The emerging antibiotic-resistant bacteria and other risk factors, including patient comorbidities, complicate patient management.

**Methods:**

A single-center retrospective observational study was conducted at King Fahad Hospital of the University, Eastern Province, Saudi Arabia. Hospitalized patients with confirmed central line-associated bloodstream infections between January 2015 and December 2020 were included. The primary objectives were to investigate the trends in antibiotic susceptibility patterns of the causative agents, coexisting comorbid conditions, and other risk factors associated with mortality.

**Results:**

A total of 214 patients with confirmed central line-associated bloodstream infections were included (CLABSI). The overall 30-day mortality rate was 33.6%. The infection rates per 1000 central line days for medical, surgical, and pediatric intensive care units were 4.97, 2.99, and 4.56 per 1000 CL days, respectively. The overall microbiological trends showed a predominance of Gram-negative agents, a steady increase of fungal CLABSI up to 24.0% in 2020, and a high prevalence of multidrug resistance up to 47% of bacterial CLABSI. In addition, the study indicates a significant negative surviving correlation with diabetes mellitus, cardiovascular disease, lung disease, chronic kidney disease, and the presence of ≥ 3 comorbidities (P < 0.05).

**Conclusion:**

The microbiological trends of the study population demonstrated a steady increase of CLABSI caused by *Candida* spp. with a predominance of Gram-negative pathogens. Stratifying the patients according to relevant mortality risk factors, including patient comorbidities, will help reduce CLABSI rates and improve patient outcomes.

## Background

Central line-associated bloodstream infection (CLABSI) is a significant cause of morbidity and mortality worldwide, associated with more extended hospital stays and higher healthcare costs [1]. The primary source of CLABSI is the contamination with microorganisms during catheter insertion, blood transfusion, drug injection, or parenteral nutrition. Additionally, endogenous source of microorganisms that may cause CLABSI should be considered. The International Nosocomial Infection Control Consortium (INICC) reported a CLABSI rate of 4.1/1000 Central Line (CL) days with a crude pool mortality of 38.4% based on data from 703 ICUs in 50 developing countries [2]. A study from six tertiary care hospitals in three Gulf countries (Bahrain, Oman, and Saudi Arabia) analyzed the data of 461 CLABSIs during six years with an overall CLABSI rate of 3.1 per 1000 CL days [3]. Another study, including 12 Ministry of Health hospitals in Saudi Arabia, reported CLABSI rates of 2.2–10.5/1000 CL days [4]. Al-Tawfiq et al. reported that after implementing a CLABSI prevention bundle in a Saudi Arabian hospital, the CLABSI incidence had been significantly reduced from 16.3 to 6.1/1000 CL days [5]. However, the burden of CLABSI fluctuates even with best practices, which indicates the need to improve prevention techniques and identify the risk factors in different patient populations [6].

Emerging healthcare-associated infections caused by bacteria nick-named “superbugs,” that have become more resistant to most available antibiotics, add another complicated dimension to the management of CLABSI. The list of multidrug-resistant organisms (MDRO) has continued to grow, with most of them belonging to the “ESKAPE” group that includes *Enterococcus faecium, Staphylococcus aureus, Klebsiella pneumoniae, Acinetobacter baumannii, Pseudomonas aeruginosa*, and *Enterobacter* spp. A study from South Korea showed a gradually increased frequency of Gram-negative bacteria for CLABSI from 24.6 to 32.6%, along with an increased resistance rate of *A. baumannii* [7]. In a study that included intensive care unit (ICU) patients from 1,791 facilities worldwide, 48.9% of *S. aureus* CLABSI isolates were MRSA, and 27.7% of *Klebsiella* CLABSI isolates were extended-spectrum cephalosporin-resistant [8]. Also, emerging resistant *Candida* species, such as *C. glabrata, C. lusitaniae, and C. auris*, are important causative agents, especially in severely ill patients, hence complicating the course of treatment of other underlying diseases with increased morbidity and mortality.

The risk factors for developing CLABSI include prolonged duration of CL, longer ICU stays, use of multiple CLs, higher APACHE scores, blood transfusion, and parenteral nutrition [9–12]. Most CLABSI prevention studies concentrate on applying different prevention strategies and measures to decrease the CLABSI rate, such as reducing CL days, assignment of a dedicated team, daily surveillance, a well-structured training program for healthcare workers inserting CLs and IV cannulations, and the use of ultrasound for CL insertion if possible [13]. However, there is a paucity of data regarding patient-related predisposing risk factors for developing CLABSI in the literature. Pepin et al. found that developing CLABSI was associated with cerebrovascular disease, chronic liver disease, and chronic renal insufficiency [14]. Harris et al., using the Delphi method in their study, have generated a list of comorbidities that should be investigated as risk factors for CLABSI and surgical site infections. This list includes but is not limited to, diabetes mellitus, dementia, drug abuse, hemiplegia or paraplegia, HIV/AIDS, malignancies, severe liver disease, and renal disease [15]. Furthermore, multiple factors that affect human immunity can cause immune dysfunction or immune dysregulation, predisposing patients to more severe infections with higher mortality rates, such as obesity, diabetes, distress, and advanced age [16, 17].

Effective and long-term CLABSI prevention requires evidence-based best practices and suitable implementation strategies that consider the procedural, microbiological, environmental, and patient factors interplaying in developing CLABSI and other Healthcare-associated infections. Hence, the objectives of this study were to (I) Determine the trends in the causative agents of CLABSI during the study period and their antibiogram, (II) Investigate the risk factors and comorbidities that may be associated with higher mortality among adult patients with CLABSI.

## Methods

### Study design, settings, and participants

We performed a single-center retrospective observational study at King Fahad Hospital of the University (KFHU), an academic tertiary medical center with 502 beds in Al-Khobar city, Eastern Province, Saudi Arabia. All included patients fulfill the inclusion criteria as per the CDC definition:

1. Patients of all age groups who were hospitalized during the study period.
2. During the hospitalization, the case developed a laboratory confirmed bacteremia after having CL insertion within the 48-hour period before the development of the infection.

The exclusion criteria were:

1. Patients with CL who developed secondary bloodstream infections related to an infection from another focus.
2. Patients who had been referred to other hospitals before completing treatment of CLABS.

The required powered sample size was 126, based on the World Health Organization calculator of sample size and our laboratory prevalence of 12% CLABSI amongst all bloodstream infections using a 95% confidence interval [18].

### Data collection

Daily infection control notes, patient hard hospital files, and electronic hospital records were reviewed to collect needed information about demographic characteristics, the indication of CL (Temporary or permanent), days of hospitalization before CLABSI, days before onset CLABSI after line insertion, type of the inserted CL (Non-tunneled, tunneled, peripherally inserted central catheter [PICC], or Port-a-Cath), lumens (single or multiple), and site (Jugular, subclavian, femoral, umbilical, or other sites). Clinical data related to underlying medical conditions, mortality at the event period, and 30-day mortality have been collected. In addition, microbiological data about causative pathogens, such as the frequency of Gram-positive, Gram-negative, and fungal CLABSI agents and antimicrobial susceptibility information of each causative agent, were also collected. The mechanisms of resistance for common pathogens were identified through automated VITEK II screening (Biomerieux, USA) and manual confirmation following the diagnostic testing methods as described by the M100 Performance Standards for Antimicrobial Susceptibility Testing including MRSA, VRE, ESBL, CRE, MDR and XDR phenotypes (CLSI 30th Edition, 2020) and the genotypic assay Genexpert carba-r, Xpert® MRSA/SA, and the BioFire BCID2 Panel. [19, 20] As per the institutional infection control policy, these phenotypes were placed under contact precautions; cohorting was only done in identical phenotypes outside the critical care settings.

### Statistical analysis

Data were collected using a Microsoft Excel sheet and then imported after cleaning for statistical analysis using the Statistical Package for Social Sciences (SPSS) version 26.0, IBM, USA. The normality of data was tested by the Shapiro-Wilk test. Categorical variables were presented as frequencies and percentages, and numerical variables as mean ± standard deviation. The Whitney U test was used for non-parametric data to compare numerical variables. Statistical analysis of categorical variables (presented as proportions) was performed using the Chi-square and Fisher’s exact tests. Significance was tested using the Pearson correlation coefficient (*r*) test to evaluate the impact/relationship between patient comorbidities and mortality and the resistance occurrence versus age and gender.

## Results

### Patients’ demographic data and CL-related information

**Fig. 1:**
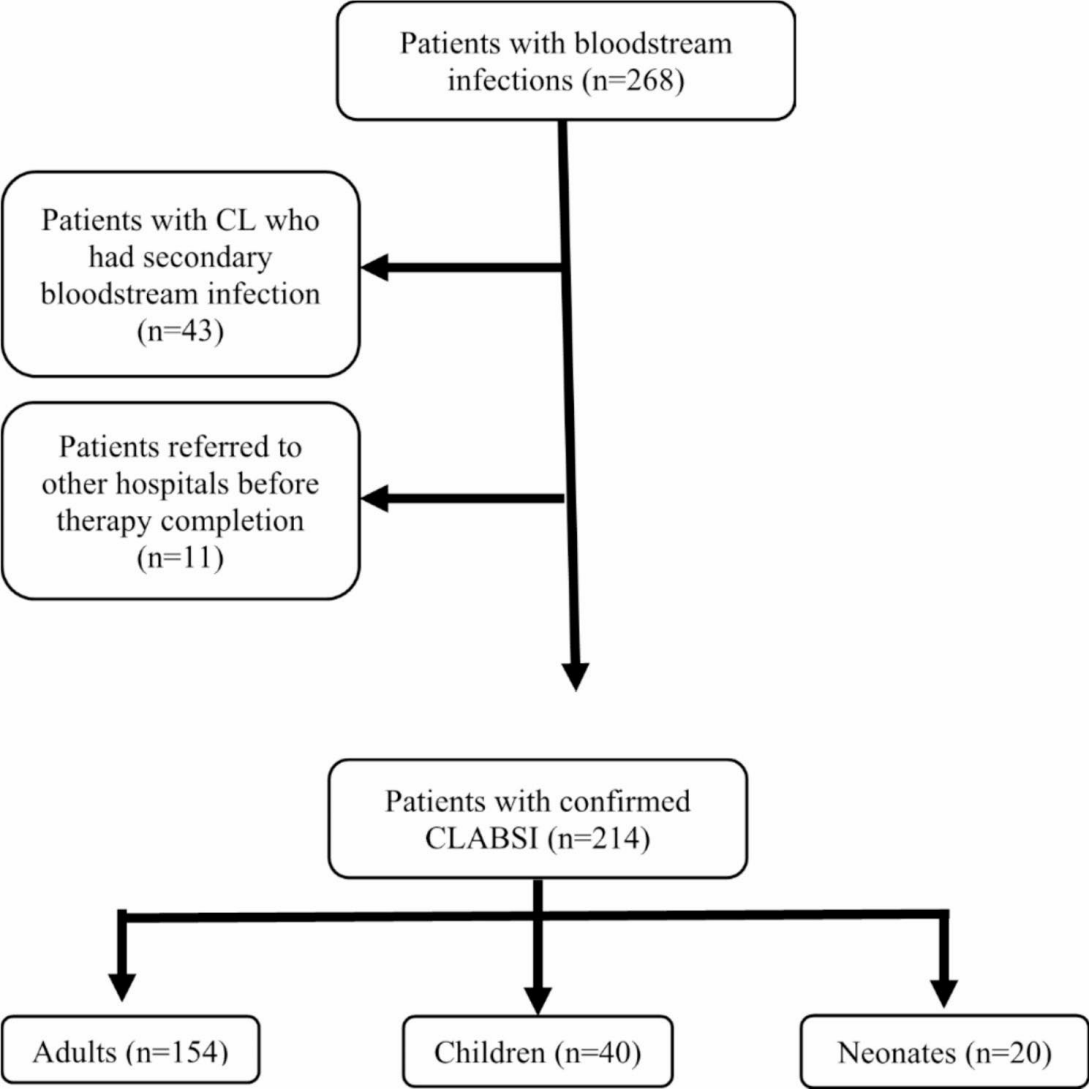
CLABSI study flow chart

A total of 214 patients with confirmed CLABSI diagnoses that fulfill the study inclusion criteria (Fig. 1) were included between 1 and 2015 and 31 December 2020, comprising 20 newborns, 40 children, and 154 adults. 71.0% of CLABSI patients were in ICUs, and 29.0% were outside ICUs. The median age was 58 (17–95) years for adults, 7 (2-168) months for children, and 17 (10–30) days for newborns. Data related to the central lines, including CL type, lumen number, indication, and site, are presented in Table 1. The non-tunneled CL was the most frequently used type, followed by PICC. The majority of central lines had multiple lumens; mean days between line insertion and CLABSI diagnosis were 17.9 days for adults, 16.8 days for children, and 12.2 days for neonates, whereas the overall 30-day mortality rate was 33.6% (38.3% for adults, 25.0% for children, and 15.0% for neonates).

**Table 1:** Patients’ demographic data, central line types, number of lumens, site, indication, and CLABSI related information

	Adults	Pediatrics	Neonates	Total
**Demographic data**				
**Gender**				
Male	97	19	9	125
Female	57	21	11	89
**Male/Female ratio**	0.62	0.475	0.450	0.584
**Median age**	58 (17–95) years	7 (2-168) months	17 (10–30) days	
**Mean age**	56.61 years	45.175 months	19 days	
**Central line type**				
Non-tunneled	115 (73%)	26 (59%)	14 (70%)	155 (70%)
Tunneled	8 (5%)	0	0	8 (4%)
Peripherally inserted central catheter (PICC)	31 (19%)	18 (41%)	6 (30%)	55 (25%)
Port-a-Cath	4 (3%)	0	0	4 (2%)
**Lumens**				
Single	8 (5%)	0	1 (5%)	9 (4%)
Multiple	150 (95%)	44 (100%)	19 (95%)	213 (96%)
**Indication**				
Temporary	145 (92%)	44 (100%)	20 (100%)	209 (94%)
Permanent	13 (8%)	0	0	13 (6%)
**Site**				
Jugular	50 (30%)	13 (30%)	0	63 (28%)
Subclavian	19 (12%)	1 (2%)	1 (5%)	21 (9%)
Femoral	55 (35%)	9 (20%)	1 (5%)	65 (29%)
Umbilical	0	2 (5%)	12 (60%)	14 (6%)
Others	34 (21%)	19 (43%)	6 (30%)	56 (27%)
**Median days of hospitalization before CLABSI diagnosis**	21 (3-1095)	32 (5-203)	15 (7–26)	
**Median Days between line insertion and CLABSI diagnosis**	9 (2-389)	14 (2–41)	12 (3–21)	
**Mean days between line insertion and CLABSI diagnosis**	17.89	16.76	12.15	
**Died at the event period (n, %)**	24 (15.58%)	4 (10.00%)	2 (10.00%)	30 (14.01%)
**30-day mortality (n, %)**	59 (38.31%)	10 (25.00%)	3 (15.00%)	72 (33.64%)

**Fig. 2:**
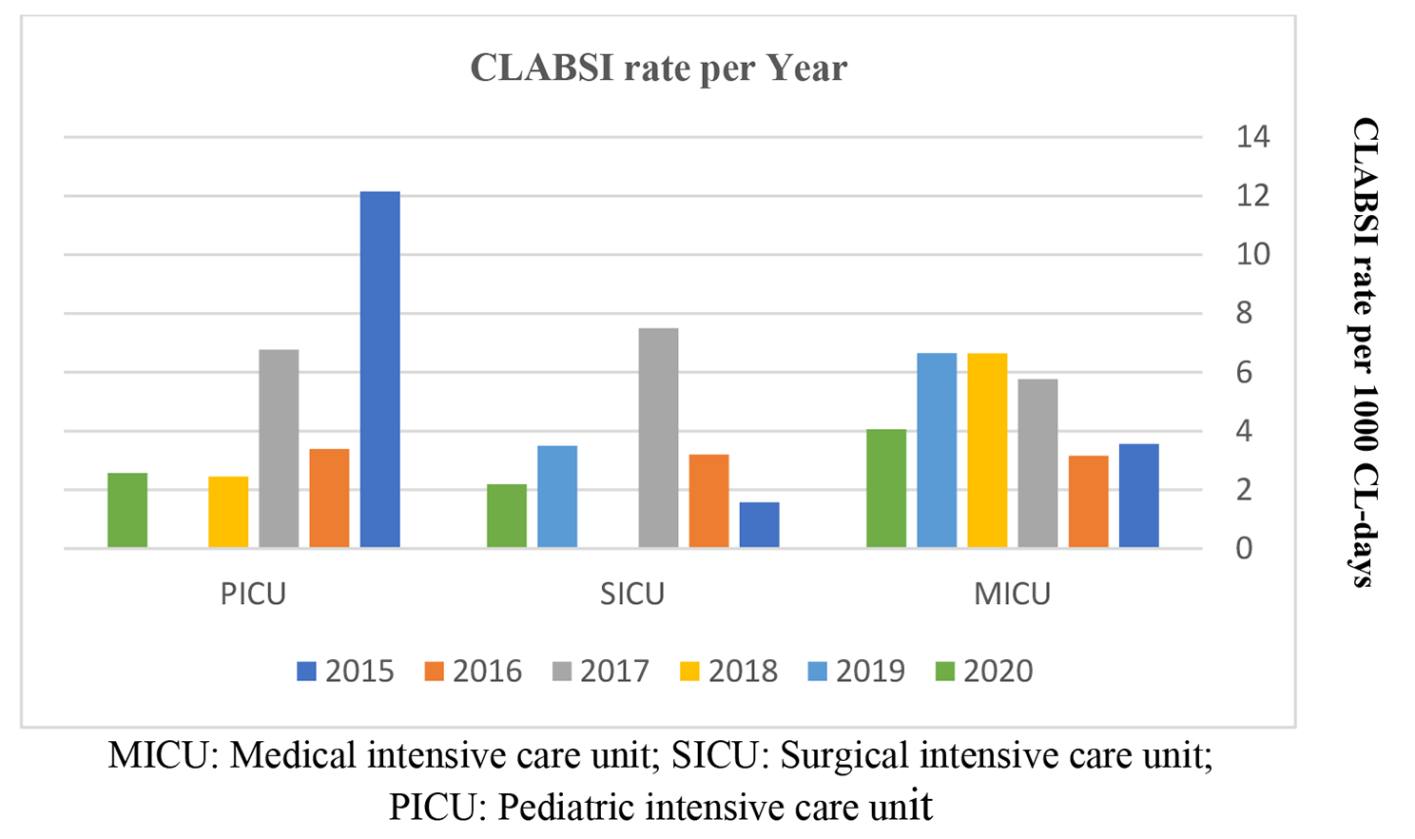
CLABSI rate per 1000 central line-days in ICUs.

The CLABSI rates per 1000 CL days for medical, surgical, and pediatric ICUs during the study period are presented in Figs. 2, 32560 CL days resulted in 125 CLABSIs with mean rates in MICU, SICU, and PICU of 4.97, 2.99, and 4.56 per 1000 CL days, respectively. In addition, the CLABSI rates per 1000 CL days were compared with the Gulf Cooperation Council (GCC) Center for Infection Control and National Healthcare Safety Network (NSHN) rates for adults and pediatric units. In terms of the possible association between CL characteristics and 30-day mortality in adults, a significantly higher mortality was observed in relation to the type of CL (P = 0.0478) and CL insertion site (P = 0.0034) (Table 2).

**Table 2:** The association between the Central line characteristics and mortality in adults

	Survivors		Non-Survivors	P-value*
**Central line type**				
Non-tunneled	98		56	P = 0.0478
Tunneled	6		2	
Peripherally inserted central catheter (PICC)	45		12	
Port-a-Cath	1		3	
**Lumens**				
Single	4		4	P = 0.4234
Multiple	96		54	
**Indication**				
Temporary	141		66	P = 0.6227
Permanent	8		5	
**Site**				
Jugular	39		24	P = 0.0034
Subclavian	14		7	
Femoral	36		28	
Umbilical	13		1	
Others	50		10	

* Chi-square test was used

## CLABSI causative agents and antimicrobial susceptibility

**Fig. 3:**
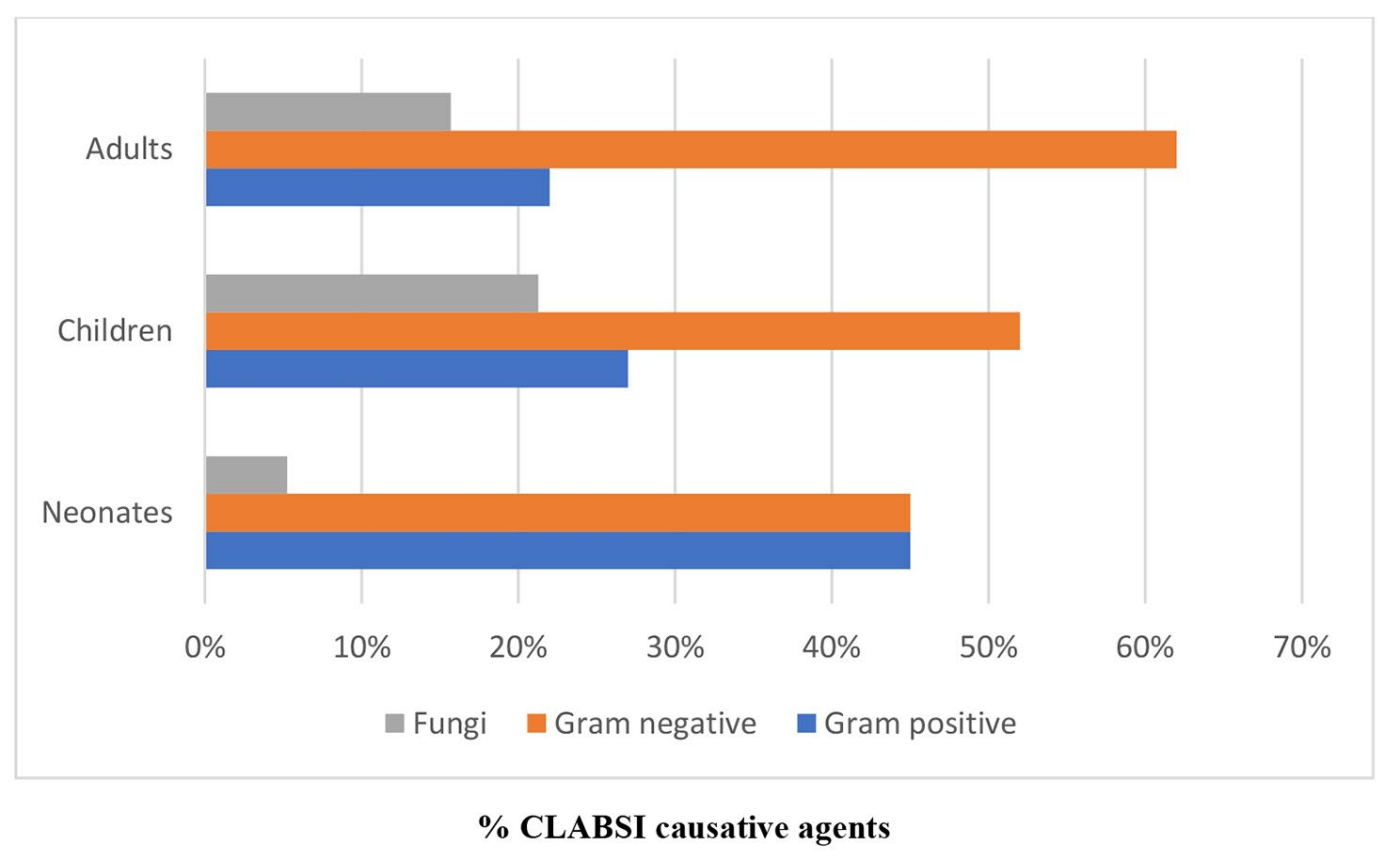
The percentage of CLABSI causative agents (Gram positive, Gram negative, and fungi) according to the patient groups

**Fig. 4:**
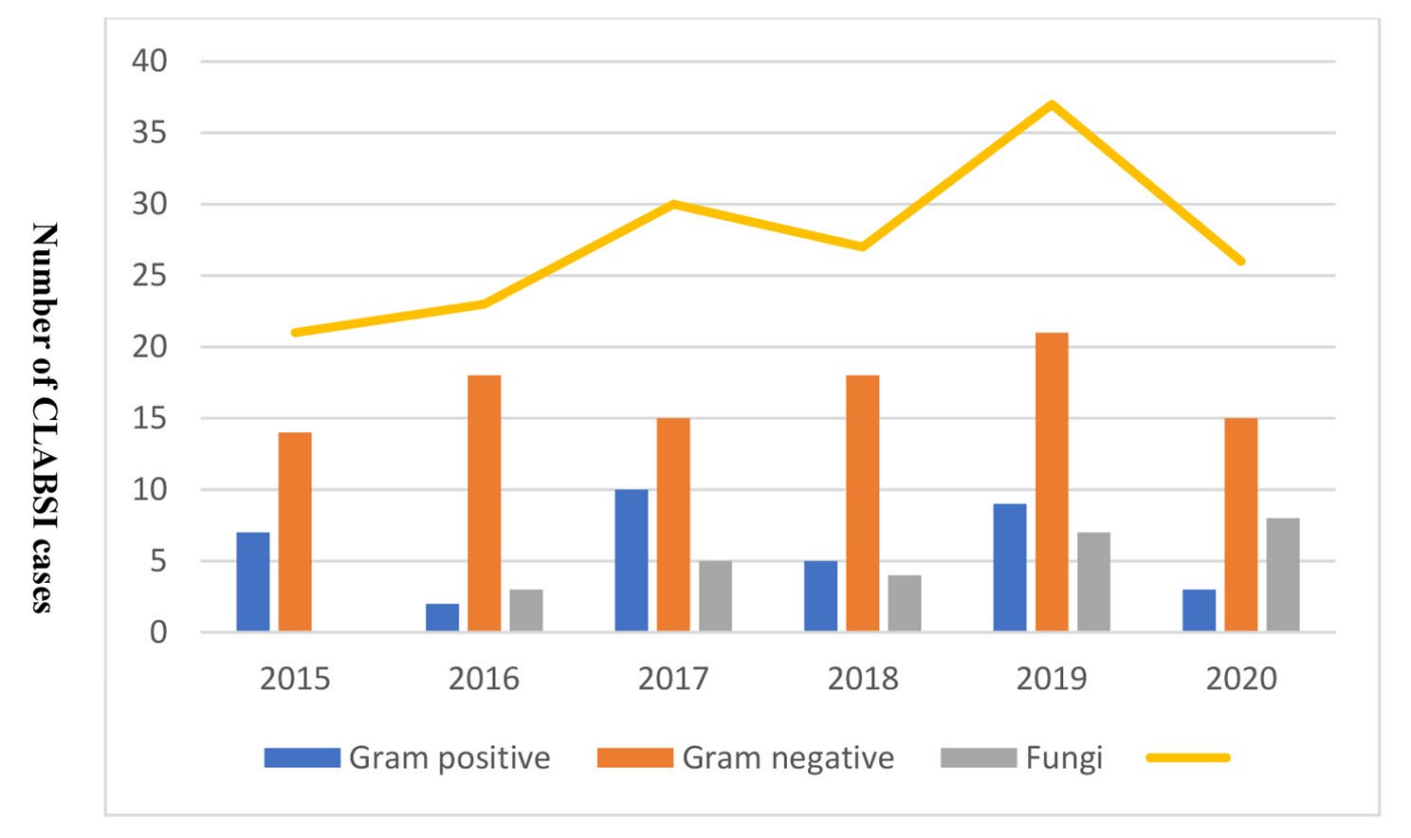
The frequency of CLABSI yearly diagnosed cases during the study period (2015–2020); the line indicates the total number of CLABSI cases diagnosed yearly

**Fig. 5:**
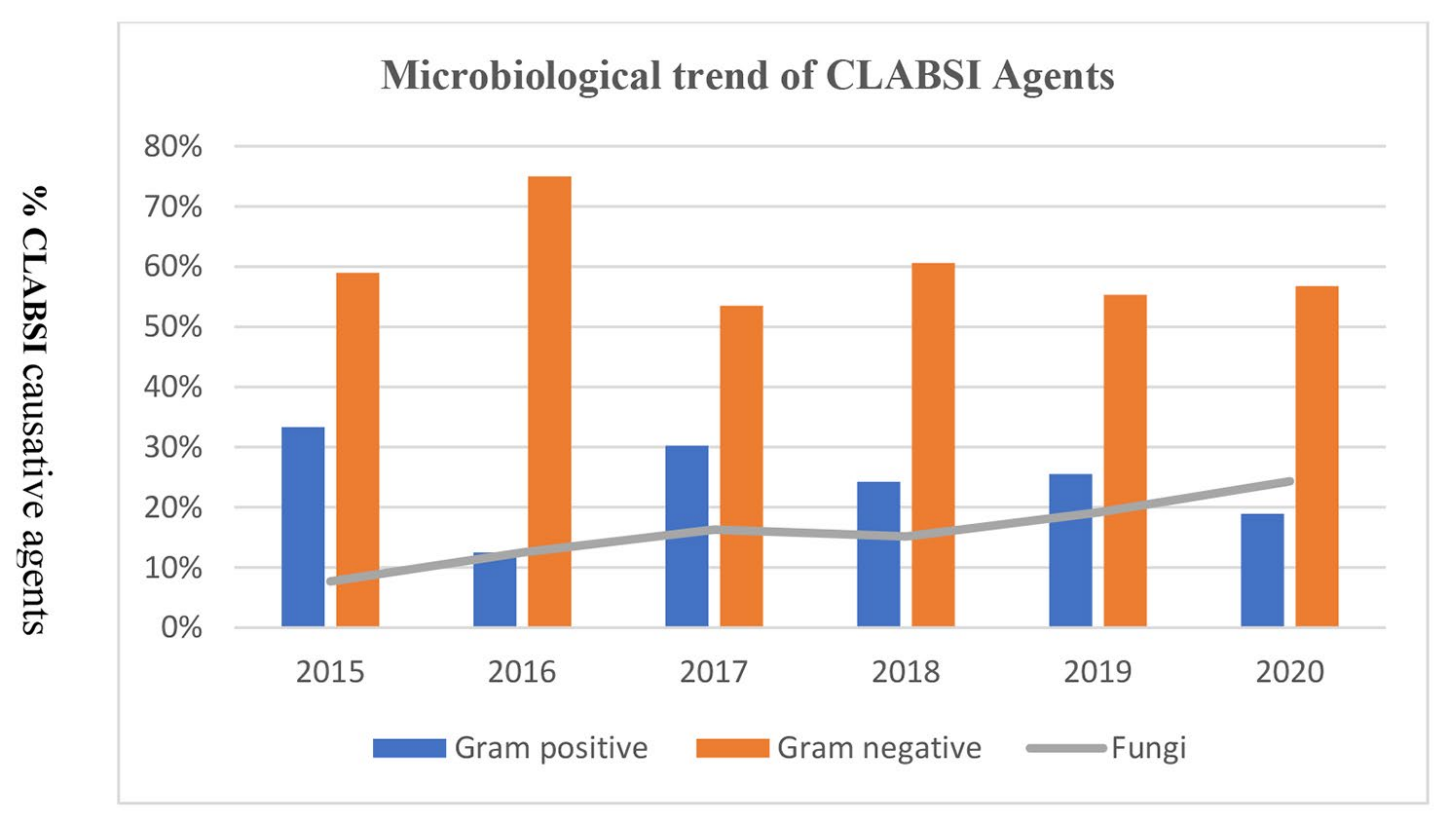
The microbiological trends of CLABSI causative agents (Gram positive, Gram negative, and fungi) during the study period (2015–2020); the line indicates fungal infections

Regarding the causative agents, Gram-negative bacteria were predominant in adults and children, whereas Gram-positive and Gram-negative bacteria had a similar rate among neonates. In addition, fungal CLABSIs were more frequent in adults and children compared with neonates; no one of the CLABSI patients caused by candida was on total parenteral nutrition; however, seven patients underwent abdominal surgeries, two had perforated viscus, and one was an oncology patient. The Gram-negative bacteria remained the dominant cause of CLABSI throughout the study period (Figs. 3 and 4). The overall microbiological trends of CLABSI causative agents showed a steady increase of fungal CLABSI from 13.0% to 2015 to 24.0% in 2020 (Fig. 5). The most frequent Gram-positive causative organism of CLABSI in the study population was Coagulase-negative *staphylococci* (11.0%), followed by *S. aureus* (9.0%). In addition, the predominant Gram-negative organisms were *Enterobacteriaceae* spp., *Acinetobacter* spp., and *Pseudomonas* spp. (42.0%, 15.0%, and 9.0%, respectively) (Table 3). Among causative fungal agents, *C. parapsilosis* was the most frequently isolated fungi (45.0% of all fungal isolates), especially in children (80.0%), followed by *C. albicans* (17.5%) (Table 3).

**Table 3:** Frequencies and percentages of different causative agents (n = 244 isolates from 214 cases)

	Adults	Children	Neonates	Total
**Gram-positive bacteria (60)**				
Coagulase-negative *staphylococci*	14 (10%)	6 (16%)	3 (16.5%)	23 (11%)
*Staphylococcus aureus*	11 (8%)	3 (8%)	5 (28%)	19 (9%)
*Enterococcus* spp.	11 (8%)	4 (10.5%)	1 (5.5%)	16 (8%)
Other Gram positives	2 (1%)	0 (0%)	0 (0%)	2 (1%)
**Gram-negative bacteria (166)**				
*Enterobacteriaceae* spp.	68 (47%)	12 (31.5%)	4 (22%)	84 (42%)
*Pseudomonas* spp.	11 (8%)	6 (16%)	1 (5%)	18 (9%)
*Acinetobacter* spp.	22 (15%)	4 (10.5%)	4 (22%)	30 (15%)
*Stenotrophomonas maltophilia*	1 (1%)	2 (5%)	0 (0%)	3 (1%)
*Burkholderia cepacia*	5 (3%)	1 (2.5%)	0 (0%)	6 (3.5%)
Other Gram negatives	1 (1%)	0 (0%)	0 (0%)	1 (0.5%)
MDR or XDR bacterium	68 (47%)	16 (41%)	8 (44%)	92 (43%)
**Fungi**: *Candida* spp. (40)				
*C. albicans*	6 (21%)	0 (0%)	1 (50%)	7 (17.5%)
*C. auris*	3 (11%)	0 (0%)	0 (0%)	3 (7.5%)
*C. glabrata*	2 (7%)	0 (0%)	0 (0%)	2 (5%)
*C. krusei*	1 (4%)	0 (0%)	0 (0%)	1 (2.5%)
*C. parapsilosis*	9 (32%)	8 (80%)	1 (50%)	18 (45%)
*C. tropicalis*	4 (14%)	1 (10%)	0 (0%)	5 (12.5%)
Other less common *Candida* spp.	3 (11%)	1 (10%)	0 (0%)	4 (10%)

There were a total of 244 isolates responsible for CLABSI in this study; the majority (204 isolates [83.6%]) were bacterial (60 Gram-positive and 144 Gram-negative) and 40 (16.4%) *candida* spp. Table 4 presents antimicrobial susceptibility patterns of the isolated organisms, which showed a significant decrease in the susceptibility of Gram-negative to wide-spectrum beta-lactam antibiotics, including carbapenems. Sixty of 204 (29.41%) bacterial isolates were multidrug-resistant; 37.0% of isolated *S. aureus* were MRSA, 61.0% of isolated *P. aeruginosa*, and 61.0% of *Acinetobacter* spp. as well were multidrug-resistant. In addition, intrinsically multidrug-resistant organisms were isolated from nine bacterial samples (6 *Burkholderia cepacia* and 3 *Stenotrophomonas maltophilia*). A total of 92 strains were classified as MDR or XDR, representing 43% of pathogens implicated in bacterial CLABSI in this cohort, with no association seen with gender or age groups (P = 0.32 and 0.57, respectively).

**Table 4:** Antimicrobial susceptibility rates of the isolated CLABSI organisms

		Susceptibility n (%)	
Organisms	Agents	*n*	(%)
**Gram positive pathogens (60)**	Beta-lactam		
	Oxacillin	17	28.3%
	Cefazolin	16	26.7%
	Ceftriaxone	16	26.7%
	Meropenem	17	28.3%
	Vancomycin	59	98.3%
	Linezolid	60	100.0%
	Rifampicin	49	81.7%
	Trimethoprim-sulfamethoxazole	30	50.0%
**Gram negative pathogens (144)**	B-Lactams		
	Ceftriaxone	70	48.6%
	Ceftazidime	98	68.1%
	Cefepime	98	68.1%
	Piperacillin-tazobactam	89	61.8%
	Meropenem	102	70.8%
	Ciprofloxacin	41	28.5%
	Trimethoprim-sulfamethoxazole	73	50.7%
	Aminoglycosides	92	63.9%

### Comorbidities and mortality risk factors

Regarding the risk factors associated with increased mortality, statistical analysis showed a significant negative surviving correlation with comorbidities, including diabetes mellitus, hypertension, cardiovascular diseases, lung diseases, renal insufficiency, and an increase in the number of associated comorbidities (≥ 3) (Table 5). On the other hand, no significant correlation was observed between the mortality and days of hospitalization prior to CLABSI or days of onset after line insertion.

**Table 5:** Associated adult CLABSI Patients comorbidities and their correlation with CLABSI patient mortality

	Survivors (*n*, %)	Non-survivors (n, %)	Total (*n*, %)	Correlation	
**DM**	33 (34.7%)	38 (64.4%)	71 (46.1%)	*r* = − 0.289	P < 0.01
**Hypertension**	46 (48.4%)	41 (69.5%)	87 (56.5%)	*r* = − 0.207	P < 0.05
**Cardiovascular diseases**				*r* = − 0.261	P < 0.01
CAD	12 (12.6%)	14 (23.7%)	26 (16.9%)	*r* = -0.144	P = 0.075
MI	0 (0.0%)	7 (11.9%)	7 (4.6%)	*r* = − 0.276	P < 0.01
HF	1 (1.1%)	3 (5.1%)	4 (2.6%)	*r* = − 0.123	P = 0.128
**Lung diseases**				*r* = -0.185	P = 0.021
BA	4 (4.2%)	4 (6.8%)	8 (5.2%)	*r* = − 0.056	P = 0.488
COPD	3 (3.2%)	3 (5.1%)	6 (3.9%)	*r* = − 0.048	P = 0.551
Other lung diseases	2 (2.1%)	7 (11.9%)	9 (5.8%)	*r* = − 0.202	P = 0.012
**Renal insufficiency**				*r* = − 0.203	P = 0.012
AKI	3 (1.7%)	3 (3.2%)	4 (2.6%)	*r* = 0.045	P = 0.582
CKD	4 (4.2%)	10 (16.9%)	14 (9.1%)	*r* = − 0.215	P = 0.007
ESRD	15(15.8%)	14(23.7%)	29 (18.8%)	*r* = − 0.099	P = 0.223
**Central nervous system (CNS) disease**				*r* = − 0.042	P = 0.607
CVA	36 (37.9%)	28 (47.5%)	64 (41.6%)	*r* = − 0.94	P = 0.245
Epilepsy	5 (5.3%)	0 (0.0%)	5 (3.2%)	*r* = 0.144	P = 0.074
Dementia	4 (4.2%)	2 (3.4%)	6 (3.9%)	*r* = 0.021	P = 0.800
Other CNS disease	7 (7.4%)	5 (8.5%)	12 (7.8%)	*r* = − 0.02	P = 0.805
**Gastrointestinal disease**				*r* = 0.047	P = 0.566
IBD	6 (6.3%)	0 (0.0%)	6 (3.9%)	*r* = 0.159	P = 0.049
Pancreatitis	1 (1.1%)	2 (3.4%)	3 (1.9%)	*r* = − 0.082	P = 0.311
Other GI diseases	6 (6.3%)	4 (6.8%)	10 (6.5%)	*r* = − 0.009	P = 0.910
**Malignancies**	3 (3.2%)	1 (1.7%)	4 (2.6%)	*r* = 0.045	P = 0.582
**Trauma**				*r* = 0.239	P < 0.01
RTA	16 (17.0%)	2 (3.4%)	18 (11.8%)	*r* = 0.206	P = 0.011
Poly trauma	14 (14.7%)	0 (0.0%)	14 (9.1%)	*r* = 0.239	P < 0.01
Head trauma	14 (14.7%)	1 (1,7%)	15 (9.7%)	*r* = 0.214	P = 0.008
**Surgical causes**				*r* = 0.199	P = 0.013
EVD or VP shunt	5 (5.3%)	0 (0.0%)	5 (3.2%)	*r* = 0.144	P = 0.074
CABAG	5 (5.3%)	3 (5.1%)	8 (5.2%)	*r* = 0.004	P = 0.962
Bariatric surgeries	6 (6.3%)	0 (0.0%)	6 (3.9%)	*r* = 0.159	P = 0.049
Perforated viscus	3 (3.2%)	1 (1.7%)	2 (2.6%)	*r* = 0.045	P = 0.582
Other surgeries	7 (7.4%)	1 (1.7%)	8 (5.2%)	*r* = 0.124	P = 0.125
**≥ 3 comorbidities**	51 (54.3%)	48 (82.8%)	99 (65.1%)	*r* = − 0.291	P < 0.01

AKI: Acute Kidney Injury; BA: Bronchial Asthma; CAD: Coronary Artery Disease; CABG: Coronary Artery Bypass Graft; CKD: Chronic Kidney Disease; CNS: Central nervous system; CVA: Cerebrovascular Accident; DM: Diabetes mellitus; ESRD: End-Stage Renal Disease; GI: Gastrointestinal; HF: Heart Failure; IBD: Inflammatory bowel disease; MI: Myocardial Infarction; RTA: Road Traffic Accident; VP: Ventriculoperitoneal. *r*: Pearson correlation coefficient

## Discussion

In the era of antibiotic resistance, emerging epidemics caused by multidrug-resistant organisms have become a big concern globally [21]. Outbreaks of hospital-acquired infections (HAIs) badly affect patient hospitalization courses and desired outcomes with increased morbidity, mortality, and healthcare costs [22, 23]. CLABSI is one of the HAIs that becomes a nightmare, especially among patients admitted to ICU and immunocompromised patients. In the literature, CLABSIs have been predominantly investigated as HAIs in ICU patients; however, the number of patients with CL in other hospital wards increases with more observed CLABSIs outside ICUs [24]. The mean days between line insertion and CLABSI diagnosis in adults, children, and neonates were 17.89, 16.76, and 12.15 days, respectively. Subha Rao et al. reported that all CL would be colonized after 11 days of insertion. In addition, Pitiriga et al. showed a steady increase in CLABSI rates with an increase in CL duration of more than ten days, which agreed with our findings [25, 26].

The mean CLABSI rates per 1000 central line days in this study were consistent with previously estimated rates in 12 Ministry of Health (MOH) Hospitals in Saudi Arabia (2.2–10.5 /1000 CL days); however, were higher than GCC Center for Infection Control and NSHN documented rates [3, 4, 27]. Our findings reflect higher CLABSI rates in MICU and PICU than rates reported in GCC countries by Balkhy et al. (3.1 per 1000 CL days) and globally by Rosenthal et al. (4.1 per 1000 CL days) [2, 3]. Moreover, the CLABSI rates in our ICUs were much higher than in the United States (0.68–0.87 per 1000 CL days) [28].

After the introduction of the CLABSI bundle and the application of best practices to prevent CLABSI development, an improvement in CLABSI rates has been documented with periods of zero rate [4]. Figure 2 shows zero rates for one year in the SICU and PICU during the study period. However, for different reasons, the CLABSI rates increased again in our ICUs and elsewhere [6]; other risk factors of CLABSI, including patient subgroups and comorbidities, should be investigated.

In general, CLABSI itself is considered an important mortality risk factor [29]; the overall 30-day mortality rate in our study was 33.64%, which is comparable with the mortality rates reported by Salgado Yepez et al. (30.3%) and Iordanou et al. (33.3%) and less than mortality rate reported in Saudi MOH hospitals (41.9%) [4, 30, 31]. A significantly lower CLABSI mortality rate was observed in patients with PICC compared with patients with non-tunneled catheters (P < 0.05), which was in line with previous studies [26, 32]. Avoiding CL insertion through the femoral site was recommended to reduce CLABSI occurrence [33]. Our data demonstrate a high prevalence of femoral CL use (29%) and increased CLABSI mortality among patients with femoral site CL insertion; however, this increase was statistically insignificant compared with jugular or subclavian sites.

Regarding the causative agents in our population, the Gram-negative bacteria remained the dominant cause of CLABSI throughout the study period (Figs. 3 and 4). The hospital microflora is usually dynamic, trending more towards Gram-negative predominance was observed in previous studies in the last two decades [7, 10, 33]. The most predominant Gram-negative organisms in our hospital were *Enterobacteriaceae* spp. (42% of all isolates), followed by *Acinetobacter* spp. (15%) and *Pseudomonas* spp. (9%), whereas Mathur et al. found *Acinetobacter* spp. The most isolated bacteria responsible for CLABSI (28.2%) [34].

Recently published data from Saudi Arabia showed, in line with our findings, the predominance of Gram-negative bacteria, with *K. pneumoniae* at the top of the list of *Enterobacteriaceae* as a causative agent of CLABSI [35]. Gram-positive bacteria were less frequently isolated in our population; Coagulase-negative *staphylococci* were found in 11% of all CLABSI-isolated bacteria, followed by *S. aureus* (9%). In contrast, the Gram-positive bacteria were, according to Al-Tawfiq et al., the leading CLABSI agents before 2010 [36].

Immunocompromised patients for different reasons and patients on total parenteral nutrition are at high risk for developing invasive fungal infections. Multiple predisposing factors for developing CLABSI by *Candida* spp. were identified according to the age groups; children with intestinal failure, gastrostomy tube, or blood transfusions are a risk group for candida-associated CLABSIs [37]. Lower birth weight of neonates is considered a risk factor for CLABSI with a high proportion of *Candida* spp. [38]. The prevalence of *Candida* spp. in CLABSI varies worldwide; Moriyama et al. reported a 4% prevalence of CLABSIs caused by *Candida* spp. [10]. The prevalence was higher in other studies; it ranged between 11.11% and 13.9% [31, 39]. Our data demonstrate a steady increase in CLABSI rate caused by *Candida* spp., from 13% to 2015 to 24% in 2020 (Fig. 5). The interplay of multiple factors might explain this trend, such as the improvement of microbiological diagnosis, overuse of broad-spectrum antibiotics, and observed increase of ICU fungal infections in the first year of COVID-19 pandemic (2020), which was the last year of the study period. According to previous studies, *C. albicans* was the most isolated type of candida in CLABSIs [34, 40]. Surprisingly, *C. parapsilosis*, a non-albicans *Candida*, was the most frequently isolated fungi. These findings, along with emerging multidrug-resistant *C. auris* in three CLABSI cases during the study period, indicate the continuous changes of the nosocomial pathogens and the selective pressure of antimicrobial agents.

Antibacterial susceptibility tests were alarming; more than 70% of Gram-positive isolates were resistant to beta-lactam antibiotics, including carbapenems. Since rifampicin is not recommended as monotherapy for Gram-positive infections, limited options remain to cover Gram-positive bacteria efficiently. The frequency of Gram-positive bacteria as CLABSI pathogens might be constant or even decreased during the study period; however, the proportion of antibiotic resistance increased, as observed in a previous study [41].

The sensitivity profiles of Gram-negative pathogens demonstrate growing resistance rates (Table 4). The sensitivity patterns of potent broad-spectrum antibiotics were not much better; around 30% were resistant to cefepime and carbapenems, whereas 38.2% were resistant to piperacillin-tazobactam. MDROs are microorganisms resistant to at least one antimicrobial agent in three or more antimicrobial categories [20]. Our findings reflect the dilemma of multidrug-resistant pathogens, classified as “superbugs” and expected to kill more than 10 million patients yearly by 2050 [42]. Sixty CLABSI bacterial isolates (29.41%) were multidrug-resistant. In addition, intrinsically multidrug-resistant organisms were isolated from nine samples. Furthermore, In a study conducted by INICC that included 50 ICUs worldwide, the resistance rates to carbapenems and amikacin were 44.3% and 29.87%, respectively [2] Salgado Yepez et al. reported 75% resistance rate of *A. baumannii* isolates to carbapenems, and more than 72.7% resistance rate of *P. aeruginosa* isolates to piperacillin-tazobactam and fluoroquinolones [30]. Our data showed emerging carbapenem-resistant *Enterobacteriaceae* that are on their way to replacing less resistant superbugs.

In the literature, several risk factors for CLABSI development have been identified; these include longer ICU stays, longer duration of CL, higher APACHE II score, parenteral nutrition, massive blood transfusion, use of corticosteroids, applying intra-aortic balloon counter-pulsation, bowel perforation, liver injury, pelvic injury, renal disease, and myocardial infarction [10, 11, 14, 43]. In turn, CLABSI is considered among the top seven causes of death in Western countries, and it has been identified as a mortality risk factor in several studies [2, 29, 44]. However, identifying the mortality risk factors in CLABSI patients was less investigated. Rosenthal et al. reported in two studies the mortality risk factors in patients diagnosed with device-associated HAI, which include older age, long stay in the ICU, female gender, and admission to oncology ICU [29, 45]. Our study findings indicate a significant negative survival correlation with other chronic illnesses, including diabetes mellitus, hypertension, cardiovascular disease, lung disease, renal insufficiency, and the presence of ≥ 3 comorbidities.

This study had considerable limitations. It was a single-center study that included three different age groups, introducing heterogeneity. In addition, as a retrospective study may suffer from a data collection bias by providing less optimal recorded information of some patients. Moreover, specific groups of patients who need advanced healthcare in spatialized centers were not represented adequately in our CLABSI populations, such as oncology patients, HIV/AIDS patients, or transplant patients. These limitations will prevent the generalization of our study findings. However, investigating the trends of CLABSI pathogens over six years and risk factors associated with mortality will help improve the CLABSI approach towards better patient-centered care and favorable outcomes.

## Conclusions

The study findings demonstrate a six-year trend of CLABSI pathogens associated with a steady increase of invasive *Candida* spp. (24%). Moreover, there is a predominance of Gram-negative pathogens that are highly multidrug-resistant. Our study indicates the need for stratification of CLABSI patients according to relevant mortality risk factors and reveals the correlation between patient comorbidities and CLABSI mortality. Further prospective studies are needed to ascertain risk factors associated with CLABSI and formulate evidence-based guidance for optimal, risk-stratified empirical therapy to improve outcomes.

### Recommendations

Based on our findings, we propose considering these morbidities along with the unit antibiogram in designing individualized empirical therapeutic regimens on a case-by-case basis, taking into account previous microbiological cultures of the patient and local epidemiology. This treatment should be tailored to be more specific once the final susceptibility pattern is obtained to prevent the emergence of more resistant clones in the units. The increased CLABSI rate caused by *Candida* spp. is alarming, necessitating strict infection control measures and improved laboratory diagnostics of fungal infections, especially in the era of drug resistance emergence and novel transmissible species like *C. auris*. Comorbidities are among the important risk factors for predicting CLABSI outcomes and should be considered before CL insertion.

## Data Availability

The data presented in this study are available on request from the corresponding author.

## Abbreviations

BSI: Bloodstream infection
CL: Central line
CLABSI: Central-line associated Bloodstream infection
GCC: Gulf Cooperation Council
HAIs: Healthcare-associated infections
ICU: Intensive care unit
INICC: International Nosocomial Infection Control Consortium
IRB: Institutional ethical review board
MDRO: Multiple drug resistance organisms
NSHN: National Healthcare Safety Network

## References

[1] Bharadwaj R, Bal A, Kapila K, Mave V, Gupta A (2014). Blood stream Infections. BioMed Res Int.

[2] Rosenthal VD, Al-Abdely HM, El-Kholy AA, AlKhawaja SAA, Leblebicioglu H, Mehta Y (2016). International Nosocomial Infection Control Consortium report, data summary of 50 countries for 2010–2015: device-associated module. Am J Infect Control.

[3] Balkhy HH, El-Saed A, Al-Abri SS, Alsalman J, Alansari H, Al Maskari Z et al. Rates of central line-associated bloodstream infection in tertiary care hospitals in 3 Arabian gulf countries: 6-year surveillance study. Am J Infect Control. 2017 1;45(5):e49–51. 10.1016/j.ajic.2017.01.027 28318648

[4] Gaid E, Assiri A, McNabb S, Banjar W (2018). Device-associated nosocomial Infection in general hospitals, Kingdom of Saudi Arabia, 2013–2016. J Epidemiol Glob Health.

[5] Al-Tawfiq JA, Amalraj A, Memish ZA (2013). Reduction and surveillance of device-associated Infections in adult intensive care units at a Saudi Arabian hospital, 2004–2011. Int J Infect Dis IJID off Publ Int Soc Infect Dis.

[6] Lissauer ME, Leekha S, Preas MA, Thom KA, Johnson SB (2012). Risk factors for central line-associated bloodstream Infections in the era of best practice. J Trauma Acute Care Surg.

[7] Choi JY, Kwak YG, Yoo H, Lee SO, Kim HB, Han SH (2015). Trends in the distribution and Antimicrobial susceptibility of causative pathogens of device-Associated Infection in Korean Intensive Care Units from 2006 to 2013: results from the Korean nosocomial Infections surveillance (KONIS) system. Open Forum Infect Dis.

[8] Soe MM, Edwards JR, Sievert DM, Ricks PM, Magill SS, Fridkin SK (2015). Evaluating state-specific antibiotic resistance measures derived from central line-associated bloodstream Infections, national healthcare safety network, 2011. Infect Control Hosp Epidemiol.

[9] Callister D, Limchaiyawat P, Eells SJ, Miller LG (2015). Risk factors for central line-associated bloodstream Infections in the era of prevention bundles. Infect Control Hosp Epidemiol.

[10] Moriyama K, Ando T, Kotani M, Tokumine J, Nakazawa H, Motoyasu A (2022). Risk factors associated with increased incidences of catheter-related bloodstream Infection. Med (Baltim).

[11] Wylie MC, Graham DA, Potter-Bynoe G, Kleinman ME, Randolph AG, Costello JM (2010). Risk factors for central line-associated bloodstream Infection in pediatric intensive care units. Infect Control Hosp Epidemiol.

[12] Torre FPFL, Baldanzi G, Troster EJ (2018). Risk factors for vascular catheter-related bloodstream Infections in pediatric intensive care units. Rev Bras Ter Intensiva.

[13] Khan R, Subhani J, Arabi Y (2019). Central line-associated bloodstream Infections in the Kingdom of Saudi Arabia. Saudi Crit Care J.

[14] Pepin CS, Thom KA, Sorkin JD, Leekha S, Masnick M, Preas MA (2015). Risk factors for central-line-associated bloodstream Infections: a focus on comorbid conditions. Infect Control Hosp Epidemiol.

[15] Harris AD, Pineles L, Anderson D, Woeltje KF, Trick WE, Kaye KS (2017). Which Comorbid conditions should we be analyzing as risk factors for Healthcare-Associated Infections?. Infect Control Hosp Epidemiol.

[16] Sabri NA, Raslan M, Shehata E, Raslan AS (2020). Depressive disorders and incidence of COVID 19: is there a correlation and management interference?. Physiol Dis Res Volume.

[17] Raslan M, Eslam MS, Sara AR, Nagwa A, Sabri NA (2022). Immune System response to COVID-19. An endless story. Acta Sci Pharm Sci.

[18] Al-Subaihi AA (2003). Sample size determination. Influencing factors and calculation strategies for survey research. Neurosciences (Riyadh).

[19] Weinstein MP. Performance standards for antimicrobial susceptibility testing: supplement M100. 30th edition. Wayne, Pa.: Clinical and Laboratory Standards Institute; 2020.

[20] Magiorakos AP, Srinivasan A, Carey RB, Carmeli Y, Falagas ME, Giske CG (2012). Multidrug-resistant, extensively drug-resistant and pandrug-resistant bacteria: an international expert proposal for interim standard definitions for acquired resistance. Clin Microbiol Infect off Publ Eur Soc Clin Microbiol Infect Dis.

[21] Arias CA, Murray BE (2009). Antibiotic-resistant bugs in the 21st Century — a clinical Super-challenge. N Engl J Med.

[22] Hensley BJ, Monson JRT (2015). Hospital-acquired Infections. Surg Oxf.

[23] Raslan M, Eslam MS, Sara AR, Nagwa AS (2020). Antimicrobial Resistance and Second Wave of COVID-19: will it impose Management Protocols deviation?. Ann Pharmacol Toxicol.

[24] Rhee Y, Heung M, Chen B, Chenoweth CE (2015). Central line-associated bloodstream Infections in non-ICU inpatient wards: a 2-year analysis. Infect Control Hosp Epidemiol.

[25] Subha Rao SD, Joseph MP, Lavi R, Macaden R (2005). Infections related to vascular catheters in a pediatric intensive care unit. Indian Pediatr.

[26] Pitiriga V, Bakalis J, Kampos E, Kanellopoulos P, Saroglou G, Tsakris A (2022). Duration of central venous catheter placement and central line-associated bloodstream Infections after the adoption of prevention bundles: a two-year retrospective study. Antimicrob Resist Infect Control.

[27] Dudeck MA, Edwards JR, Allen-Bridson K, Gross C, Malpiedi PJ, Peterson KD (2015). National Healthcare Safety Network report, data summary for 2013, device-associated Module. Am J Infect Control.

[28] Patel PR, Weiner-Lastinger LM, Dudeck MA, Fike LV, Kuhar DT, Edwards JR (2022). Impact of COVID-19 pandemic on central-line–associated bloodstream Infections during the early months of 2020, National Healthcare Safety Network. Infect Control Hosp Epidemiol.

[29] Rosenthal VD, Jin Z, Memish ZA, Daboor MA, Al-Ruzzieh MA, Hussien NH (2022). Risk factors for mortality in ICU patients in 10 middle eastern countries: the role of healthcare-associated Infections. J Crit Care.

[30] Salgado Yepez E, Bovera MM, Rosenthal VD, González Flores HA, Pazmiño L, Valencia F (2017). Device-associated Infection rates, mortality, length of stay and bacterial resistance in intensive care units in Ecuador: international nosocomial Infection Control Consortium’s findings. World J Biol Chem.

[31] Iordanou S, Middleton N, Papathanassoglou E, Raftopoulos V (2017). Surveillance of device associated Infections and mortality in a major intensive care unit in the Republic of Cyprus. BMC Infect Dis.

[32] Lv Y, Huang X, Lan Y, Xia Q, Chen F, Wu J (2022). Peripherally inserted central catheters have a protective role and the effect of fluctuation curve feature in the risk of bloodstream Infection compared with central venous catheters: a propensity-adjusted analysis. BMC Infect Dis.

[33] Pitiriga V, Kanellopoulos P, Bakalis I, Kampos E, Sagris I, Saroglou G (2020). Central venous catheter-related bloodstream Infection and colonization: the impact of insertion site and distribution of multidrug-resistant pathogens. Antimicrob Resist Infect Control.

[34] Mathur P, Khurana S, Kumar S, Gupta D, Aggrawal R, Soni KD (2021). Device associated Infections at a trauma surgical center of India: Trend over eight years. Indian J Med Microbiol.

[35] Saleem M, Syed Khaja AS, Hossain A, Alenazi F, Said KB, Moursi SA (2023). Pathogen Burden among ICU patients in a Tertiary Care Hospital in Hail Saudi Arabia with Particular Reference to β-Lactamases Profile. Infect Drug Resist.

[36] Al-Tawfiq JA, Abed MS (2009). Prevalence and antimicrobial resistance of health care associated bloodstream Infections at a general hospital in Saudi Arabia. Saudi Med J.

[37] Klatte JM, Newland JG, Jackson MA (2013). Incidence, classification, and risk stratification for Candida Central Line–Associated Bloodstream Infections in Pediatric patients at a Tertiary Care Children’s hospital, 2000–2010. Infect Control Hosp Epidemiol.

[38] Crivaro V, Bogdanović L, Bagattini M, Iula VD, Catania M, Raimondi F (2015). Surveillance of healthcare-associated Infections in a neonatal intensive care unit in Italy during 2006–2010. BMC Infect Dis.

[39] He Y, Zhao H, Wei Y, Gan X, Ling Y, Ying Y (2019). Retrospective analysis of Microbial colonization patterns in central venous catheters, 2013–2017. J Healthc Eng.

[40] Pfaller MA, Jones RN, Doern GV, Fluit AC, Verhoef J, Sader HS (1999). International surveillance of blood stream Infections due to Candida species in the European SENTRY Program: species distribution and antifungal susceptibility including the investigational triazole and echinocandin agents. SENTRY Participant Group (Europe). Diagn Microbiol Infect Dis.

[41] Laupland KB, Lyytikäinen O, Søgaard M, Kennedy KJ, Knudsen JD, Ostergaard C (2013). The changing epidemiology of Staphylococcus aureus bloodstream Infection: a multinational population-based surveillance study. Clin Microbiol Infect off Publ Eur Soc Clin Microbiol Infect Dis.

[42] Honigsbaum M (2018). Superbugs and us. Lancet Lond Engl.

[43] Lee HJ, Choi E, Choi NJ, Sun HW, Lee JS, Lee JW (2020). Risk factors of Bacteremia following multiple traumas. Emerg Med Int.

[44] Goto M, Al-Hasan MN (2013). Overall burden of bloodstream Infection and nosocomial bloodstream Infection in North America and Europe. Clin Microbiol Infect off Publ Eur Soc Clin Microbiol Infect Dis.

[45] Rosenthal VD, Jin Z, Rodrigues C, Myatra SN, Divatia JV, Biswas SK et al. Risk factors for mortality over 18 years in 317 ICUs in 9 Asian countries: the impact of healthcare-associated Infections. Infect Control Hosp Epidemiol. 2022;1–6. 10.1017/ice.2022.245 36278508

